# Premature closure underlies bias in medical diagnosis in students: A randomised controlled experiment

**DOI:** 10.1111/medu.70229

**Published:** 2026-04-28

**Authors:** Awad Al Essa, Henk G. Schmidt, Silvia Mamede, Karla Knežević, Laura Zwaan, Mohi Magzoub

**Affiliations:** ^1^ United Arab Emirates University Al Ain United Arab Emirates; ^2^ Erasmus University Rotterdam the Netherlands; ^3^ The University of Amsterdam Amsterdam the Netherlands

## Abstract

**Objective:**

The purpose of the study reported in this article was to shed light on the cognitive mechanism mediating between biasing information and diagnostic error. The literature suggests at least two different hypotheses: premature closure leading biased participants to spend *less* time on diagnosis or increased competition between diagnostic hypotheses. The latter hypothesis predicts that biased participants would spend *more* time reaching a diagnosis.

**Method:**

Using the salient distracting findings (SDF) experimental paradigm, we biased 58 fourth‐year medical students while diagnosing 12 clinical vignettes in a within‐group incomplete block design under three conditions: cases presented without SDF, with SDF at the beginning and with SDF at the end. For each of these conditions, diagnostic accuracy, the number of SDF‐related mistakes and time per word needed to process the case were recorded. The data were analysed using linear mixed modelling. Estimated marginal mean scores were reported.

**Results:**

Participants confronted with salient distracting features (SDFs) at the beginning of a clinical case demonstrated significantly lower diagnostic accuracy (mean 0.11) compared with the No‐SDF condition (0.27), representing a 61% reduction (F_2,693_ = 11.995, p < 0.001), and made more SDF‐related mistakes (F_2, 693_ = 16.395, p < 0.001). When SDFs were presented at the end of the case, diagnostic accuracy was also reduced (mean 0.17; 36% reduction), but processing time did not differ from the No‐SDF condition. Only early presentation of SDFs was associated with reduced processing time per word (F_2,636_ = 4.799, p < 0.01), consistent with premature closure.

**Conclusion:**

These findings demonstrate that biasing information increases diagnostic error in medical students and that only early bias is associated with reduced information processing. The data do not support the competition hypothesis for early bias, as processing time did not increase under biasing conditions. Premature closure can therefore be directly observed rather than inferred, inviting further research.

## Abstract 2

This new study demonstrates that premature closure—deciding too early—can be directly measured and explains how bias leads to diagnostic errors. #MedEd

## 1 INTRODUCTION

The literature on diagnostic bias in medicine is extensive, with over 25 000 studies identified in Web of Science. More than 30 types of biases have been described, emerging from three main sources: (1) patient history cues that prompt irrelevant diagnoses,^1^, ^2^, ^3^ (2) situational influences such as prior patient encounters^4^, ^5^, ^6^, ^7^ and (3) physician traits like overconfidence or risk aversion.^8^, ^9^ Experimental studies consistently show their negative effect on diagnostic accuracy.^10^

Most research on cognitive responses to bias relies on dual process theory,^11^, ^12^ which contrasts fast, intuitive reasoning with slower, analytical thinking. Although Kahneman argued that errors stem mainly from the fast system,^13^ later work revealed that both systems are error‐prone, limiting the theory's explanatory power for diagnostic mistakes.^12^, ^14^, ^15^

Beyond dual process theory, two alternative mechanisms have been proposed to explain how bias leads to diagnostic error. Premature closure refers to the early acceptance of a diagnostic hypothesis, followed by curtailed consideration of alternatives, whereas cognitive conflict implies prolonged reasoning due to competition between multiple hypotheses. Although both mechanisms have been invoked in the literature, empirical work has rarely distinguished between them directly, and premature closure has typically been inferred rather than measured.

This study investigates two alternative mechanisms: premature closure, where early biased diagnoses reduce further consideration of alternatives,^16^ and cognitive conflict, where competing hypotheses prolong processing.^17^ Using Mamede's salient distracting feature (SDF) paradigm,^3^, ^18^ clinical vignettes with or without SDFs were presented at different points in the case. Processing time per word was measured across history, examination and lab results. We hypothesised that early SDFs would shorten processing (premature closure), while SDF‐induced conflicts would lengthen it.

A secondary aim was to assess bias sensitivity in medical students.^6^, ^7^, ^19^ Fifty‐eight fourth‐year students diagnosed 12 cases under three conditions (no SDF, SDF at start, SDF at end). Diagnostic accuracy, SDF‐related errors and processing time were analysed using linear mixed modelling.

## 2 METHODS

### 2.1 Participants

One‐hundred and five fourth‐year preclinical medical students from a federal university in the United Arab Emirates were invited to participate in the experiment on 15 September 2023. Eventually, 58 of them participated: 45 females and 13 males. Their mean age was 20.59 years (*SD* = 0.73). The sample size was computed based on previous studies demonstrating that mean differences tend to revolve around 0.15 with a mean standard deviation of 0.20.^3^, ^18^, ^20^ The sample size can then be computed with the formula

$$n=1+2C\left(\frac{s}{d}\right)2$$

where *s* is the standard deviation to be expected and *d* is the difference between means. *C* is a constant based on α‐level and power of the test. With an α < 0.05 and power = 0.80, C = 7.85. From this, it follows that n should be at least equal to 29. This suggests that our sample was sufficiently large to find treatment effects if they exist. Participation was voluntary, without any financial compensation. All the participants consented to be included in the data analysis. Since the nature of the study prevented prior disclosure of its objectives, participants were debriefed afterwards. The study has been reviewed and approved by the United Arab Emirates University Social Sciences Ethics Committee, reference ERSC_2023_3011.

### 2.2 Materials

The materials consisted of 12 clinical vignettes that were prepared by two board‐certified internists based on real patients and had a confirmed diagnosis. They had been used in previous experiments, and therefore, their ability to produce bias was known.^3^, ^18^ In the three versions of the experiment, participants are randomly assigned to one of the three versions, shown in a presentation of the vignettes to the participants within version in random order as stated in Appendix A. Each vignette consisted of a brief description of a patient's medical history, present complaints, symptoms and findings from physical examination and diagnostic tests. Table 1 contains an example of such case. The SDF information is underlined.

**TABLE 1:** Example of a clinical vignette used in the experiment. The SDF information appears here in the disease history and is underlined.

A 45‐year‐old lawyer complains of persistent pain in the upper abdomen. According to the patient, the pain is due to stress due to the decline in client numbers and his divorce 2 years ago. He says he was operated on for prostate cancer 5 years ago; there have been no recent studies of disease activity. Two years ago, he was treated for a duodenal ulcer. He occasionally visits prostitutes but was found to be impotent the last time. He has smoked 40 cigarettes a day for many years and has consumed significant amounts of alcohol. There is no evidence of intolerance to certain foods **Physical examination:** Spider nevi on the thorax. Abdomen: The abdomen is somewhat distended without abnormal percussion. Liver is palpable with an irregular surface. The spleen is not enlarged. Extremities: Ankle oedema 1+. Testicles: Greatly reduced **Laboratory tests:** Hb 5.0 mmol/L; BSE 44 mm/h; Na 138 mmol/L; K 3.6 mmol/L; ALAT 120 U/L; ASAT 84 U/L; LDH 800 U/L; y‐GT 250 U/L; alk. phosph. 200 U/L; bilirubin 42.7 μmol/L

The clinical cases consisted of diseases that are frequently seen or atypical presentations of frequent diseases. Table 2 contains the list of diseases diagnosed by the students.

**TABLE 2:** List of diseases to be diagnosed.

Stomach cancer Aortic dissection Addison's disease **Meningoencephalitis** Liver cirrhosis Inflammatory bowel disease Acute bacterial endocarditis Hyperthyroidism Vitamin B12 deficiency Coeliac disease Acute appendicitis Small cell lung cancer

The clinical vignettes appeared in three versions: without the SDF, with the SDF at the beginning and with the SDF at the end.

The vignettes were presented in random order on a computer screen using the survey tool Qualtrics (https://www.qualtrics.com/) with the following instruction:

Clinical reasoning is a very important skill a doctor must acquire. This research project aims to better understand the nature of this skill, vital to the well‐being of patients. Please note that your responses are anonymous since no identifying information will be collected, and your results will have no implications for your work.

Your task is to diagnose the clinical cases that will be presented to you shortly. All cases are based on real patients and have a confirmed diagnosis. It helps if you actively imagine yourself in a busy emergency room. There are a large number of patients to be seen during the rest of your shift, and only very limited time left. Try to work as quickly as possible; the first impression is often correct. Do this, of course, without compromising accuracy.

The different sections of each clinical case, patient history, physical examination findings and laboratory test results, were presented on separate screens. Once the student indicated that they had sufficiently processed the information from the screen and continued to the next screen. This was done to model the actual encounter with a patient (in which information is also shared sequentially) within the limitations of an experimental context. After completing a case, they were asked to provide a most likely diagnosis before continuing to the next case. Time spent on each of the sections was recorded. The participants were free to use as much time as needed. Before engaging in the experiment, they diagnosed a practice case to familiarise them with the procedure.

The experiment was set up as a within‐subject incomplete block design. A complete block design means that all participants receive all versions of the stimulus materials. For the present experiment, a complete block design would have implied that all students would receive all three versions of the same clinical cases. Under such conditions, students would likely learn from previous encounters with the same case. Therefore, we used an incomplete block design as exemplified by Appendix A. In an incomplete block, participants encounter similar stimuli materials only once. Every student was randomly assigned to each of the three conditions albeit with a different set of four clinical cases.

### 2.3 Data analysis

#### 2.3.1 Diagnostic accuracy

We first classified the diagnoses provided by the participants according to their accuracy. All participants responses were reviewed by two independent experts, each of the reviewers classified the diagnosis based on the same criteria provided to them. They used the confirmed diagnosis of each case to evaluate the diagnoses given by the participants as fully correct, (1) partially correct (0.5), or incorrect (0). A diagnosis was considered correct whenever the main component of the diagnosis (i.e. the main/core diagnosis) appears in the diagnosis indicated by the participant. When one of the constituent elements of the diagnosis appears in the diagnosis written down by the participant, but the core diagnosis was not cited, they judged it as partially correct. Finally, responses that did not fall into one of these categories were classified as incorrect. For example, for the case of liver cirrhosis in Table 1, ‘cirrhosis’ was judged as correct, ‘liver abnormality’ as partially incorrect, and ‘hyperlipidaemia’ as incorrect. They scored the participants' responses according to a scoring grid that was prepared by experts in internal medicine, based on the scoring of diagnoses that were provided by participants on the same cases in previous studies. The two judges showed sufficient inter‐rater agreement (intraclass coefficient [ICC] = 0.79), and in the case of discrepancies, a final score was discussed and agreed upon. Diagnostic accuracy was computed as a mean score for each participant for each condition of the experiment.

#### 2.3.2 SDF‐related diagnostic mistakes

In subsequent analysis, the judges classified the *incorrect* diagnoses as either associated with the SDF (i.e. the disease with which the SDF was strongly associated) or not associated with the SDF (i.e. other errors not likely to result from the SDF). For each case, there was a list of diagnoses that would be considered associated with the SDF based on previous research, and participants' responses were classified according to this list. For instance, for the liver cirrhosis case displayed in Table 1, ‘recurrent prostate cancer’ was considered an SDF‐related error. The ICC for this analysis was 0.91. SDF‐related error was expressed as a mean score for each participant for each condition of the experiment.

#### 2.3.3 Processing time

Time spent was divided by the number of words in the case to control for differences in text length. This was done for the case as a whole and for each section of the case (patient history, physical examination and laboratory test results). The resulting scores are thus expressed as seconds per word.

## 3 RESULTS

Table 3 presents estimated means, standard errors and 95% confidence intervals for diagnostic accuracy.

**TABLE 3:** Estimated means, standard errors and 95% confidence intervals for diagnostic accuracy.

Condition	Mean	SE	95% CI	
No SDF	0.269	0.023	0.224	0.315
SDF at the beginning	0.110	0.023	0.064	0.156
SDF at the end	0.168	0.023	0.122	0.214

The effect of the presence of the SDF on diagnostic accuracy was significant, F_2,693_ = 11.995, p < 0.001. Pairwise contrasts between conditions showed the dependent variables to differ significantly from each other (except for the contrast between accuracy under SDF at the beginning and at the end). These findings demonstrate that the participants' diagnostic accuracy suffered when SDFs were present in the clinical cases. Diagnostic accuracy decreased by 61% when an SDF was present at the beginning and by 36% when it was present at the end. The participants were clearly biased by the presence of salient distracting findings.

Table 4 contains presents estimated means, standard errors and 95% confidence intervals for SDF‐related errors.

**TABLE 4:** Estimated means, standard errors, and 95% confidence intervals for SDF‐related errors.

Condition	Mean	SE	95% CI	
No SDF	0.129	0.027	0.077	0.182
SDF at the beginning	0.330	0.027	0.277	0.382
SDF at the end	0.302	0.027	0.249	0.354

The effect of the presence of the SDF on SDF‐related mistakes was significant, F_2,693_ = 16.395, p < 0.001. Pairwise contrasts showed the mean proportion of mistakes under the No‐SDF condition to be significantly different from the other two conditions. The mean proportions of mistakes made under the two SDF conditions did not significantly differ. SDF‐related errors increased by around 60% when SDFs were present in the case. These findings provide evidence that the mistakes made are not random errors stemming from general confusion, but the result of the biasing information.

Linear mixed modelling was also conducted on total processing times per word. Table 5 contains the relevant means, standard errors and 95% confidence intervals.

**TABLE 5:** Estimated means, standard errors and 95% confidence intervals for total processing times per word.

Condition	Mean	SE	95% CI	
No SDF	0.490	0.023	0.445	0.535
SDF at the beginning	0.436	0.023	0.391	0.482
SDF at the end	0.463	0.023	0.418	0.509

The effect of the presence of the SDF on total processing time per word was significant, F_2,636_ = 4.799, p < 0.01. Pairwise contrasts showed mean time under the SDF‐at‐the‐beginning condition to be significantly *less* than under the No‐SDF condition. Time spent while the SDF appeared at the end did not significantly differ from the No‐SDF condition. These findings clearly support a premature closure effect of biasing information on the processing of clinical vignettes. A detailed breakdown of processing time by case section (history, physical examination and laboratory tests) is presented in Table 6.

**TABLE 6:** Estimated means for processing times per word for history, physical examination and laboratory test sections.

Condition	History	Physical examination	Laboratory tests
No SDF	0.483	0.486	0.538
SDF at the beginning	**0.434**	**0.410**	0.480
SDF at the end	0.474	0.466	0.449
Test of significance	F_2,636_ = 3.895, p < 0.03	F_2,636_ = 4.288, p < 0.02	F_2,636_ = 2.003, non‐significant

These results, however, mirror largely the total time per word findings. In fact, the relevant processing times tend to be *smaller* when an SDF is present (see the findings in bold), again pointing at a premature closure interpretation.

## 4 DISCUSSION

The purpose of the study reported in this article was to shed light on the cognitive mechanisms mediating between biasing information and diagnostic error. To that end, we asked medical students to diagnose 12 clinical vignettes under three experimental conditions in a within‐group design. The cases under the SDF‐at‐the‐beginning condition contained salient distracting findings early in the text. In addition, an SDF‐at‐the‐end condition and a No‐SDF condition were included. We measured diagnostic accuracy, SDF‐related mistakes and time per word spent on processing the clinical cases. We found the experimental manipulation to be successful: The participants' diagnostic accuracy significantly decreased, and SDF‐related errors increased under the biasing conditions. These findings are in line with previous studies involving residents.^3^, ^18^

More importantly, we found that participants under the SDF‐at‐the‐beginning condition generally spent *less* processing time (but not under the SDF‐at‐the‐end condition). Hence, time invested in processing clinical case information seems to play an important mediating role between biasing events and diagnostic error.

Why did participants spend less time processing the cases under the SDF‐at‐the‐beginning condition but not under the other conditions? Participants also made substantially more SDF‐related diagnostic mistakes under this condition, suggesting that early salient information exerted a strong influence on diagnostic reasoning. One plausible interpretation is that participants accepted an initially plausible but incorrect diagnostic hypothesis and invested less effort in processing subsequently presented information, a pattern consistent with premature closure.

However, reduced processing time does not uniquely identify premature closure. Alternative mechanisms may also account for this pattern. For example, early exposure to a SDF may have triggered confirmation bias, whereby participants continued to process subsequent information primarily to verify an initial hypothesis rather than to evaluate alternatives. Thus, while the observed reduction in processing time is consistent with premature closure, it cannot be taken as definitive evidence that this mechanism operated in isolation.

Premature closure has long been recognized as a major contributor to diagnostic errors.^16^, ^21^, ^22^, ^23^, ^24^, ^25^ However, to the extent that an attempt was made to *measure* premature closure in relevant studies, it was done indirectly, for instance, by pointing at incomplete problem lists as an indicator of premature closure,^25^, ^26^ by advocating the compilation of a full differential diagnosis to avoid premature closure^27^ or by suggesting that the use of checklists may decrease premature closure.^28^, ^29^ What premature closure as an empirical phenomenon entails has remained, however, largely elusive. We believe that our study is the first in which premature closure is measured directly, namely, as a decreased investment of time to process a clinical case in response to an early wrong but salient diagnostic hypothesis.

## 5 CONCLUDING REMARKS

In this study, we have demonstrated that premature closure can mediate between biasing information and diagnostic error. Importantly, the findings do not support the competition hypothesis for early bias, as biased participants did not spend more time processing the cases. Instead, reduced processing time was observed only when biasing information was presented early, consistent with premature closure. We have further demonstrated that premature closure can be directly observed rather than simply assumed, as is often the case in existing literature. Further research is necessary to assess whether, as suspected by Graber and colleagues, premature closure is also the intermediary cognitive process underlying negative effects on patient safety of other biases.

Premature closure is sometimes considered a cognitive bias itself.^30^, ^31^, ^32^ We disagree. Premature closure is rather a consequence of the confidence that physicians attach to an early but biased diagnostic hypothesis. The biasing information is the culprit, not premature closure.

Several procedures have been proposed to prevent premature closure. Among them are checklists,^29^ cognitive debiasing,^32^, ^33^ reconsidering initial diagnoses^13^ or artificial intelligence.^24^ While the extant literature emphasises the importance of faulty reasoning strategies stimulated by the work of Tversky and Kahneman,^34^ we would like to point out the *lack of knowledge* as a basis for susceptibility to bias. Elsewhere, we have demonstrated that effects of bias do not emerge when physicians have sufficient appropriate knowledge of the diseases under scrutiny, in particular knowledge of findings discriminating between look‐alike diseases.^20^, ^35^, ^36^ See also Norman et al.^12^, ^14^

The study has several limitations. The use of clinical vignettes rather than real patient encounters is one of them. However, clinical vignettes have been demonstrated to be valid instruments for measuring diagnostic accuracy.^37^, ^38^ A second and more important limitation is that the study was conducted with advanced students rather than with physicians. Students might respond to the cases and particularly to the biasing information differently than more experienced physicians. However, the findings regarding diagnostic accuracy and SDF‐related mistakes under the three conditions, as exemplified in Tables 3 and 4 are similar to those among residents in earlier studies.^3^, ^18^

An additional limitation concerns the explicit instruction to work as quickly as possible and to rely on first impressions. Although this instruction was intended to simulate time pressure in clinical environments, it may also have increased participants' susceptibility to premature closure or other biases. This instruction could therefore have amplified the observed effects, and future studies should examine whether similar patterns emerge under less time‐pressured conditions.

## AUTHOR CONTRIBUTIONS

**Awad Al Essa:** Project administration; resources; writing—review and editing; investigation; data curation; visualization. **Henk G. Schmidt:** conceptualization; investigation; validation; writing—review and editing; writing—original draft; project administration; supervision; methodology; formal analysis. **Silvia Mamede:** Formal analysis; validation; investigation; writing—review and editing. **Karla Knežević:** Conceptualization; writing—review and editing; writing—original draft; formal analysis; software. **Laura Zwaan:** Software; investigation. **Mohi Magzoub:** Validation; writing—original draft; writing—review and editing; formal analysis; project administration; resources; visualization; conceptualization.

## CONFLICT OF INTEREST STATEMENT

The authors declare no conflicts of interest.

## ETHICS STATEMENT

The study has been reviewed and approved by the United Arab Emirates University Social Sciences Ethics Committee, reference ERSC_2023_3011.

## Data Availability

The data that support the findings of this study are available on request from the corresponding author. The data are not publicly available due to privacy or ethical restrictions.

## Appendix A

Three versions of the experiment. Participants randomly assigned to one of the three versions, presentation of the vignettes to the participants within version in random order.

**Table:**

	Without SDF	With SDF at the beginning	With SDF at the end
Version 1	Vignette 1	Vignette 2	Vignette 3
	Vignette 4	Vignette 5	Vignette 6
	Vignette 7	Vignette 8	Vignette 9
	Vignette 10	Vignette 11	Vignette 12
Version 2	Vignette 2	Vignette 3	Vignette 4
	Vignette 5	Vignette 6	Vignette 7
	Vignette 8	Vignette 9	Vignette 10
	Vignette 11	Vignette 12	Vignette 1
Version 3	Vignette 3	Vignette 4	Vignette 5
	Vignette 6	Vignette 7	Vignette 8
	Vignette 9	Vignette 10	Vignette 11
	Vignette 12	Vignette 1	Vignette 2
